# Meteorological Table, for August

**Published:** 1854-09

**Authors:** Lawrence Young


### METEOROLOGICAL TABLE, FOR AUGUST.

BY LAWRENCE YOUNG, ESQ.

| Day of month. | Morning. | 2 o'clock, P. M. | Evening. | Mean temperature. | Barometer. | Rain-gauge. | Course of wind. | Remarks. |
|---|---|---|---|---|---|---|---|---|
| 1 | 72 | 100 | 91 | 88 | 29.68 | | w. | Variable. |
| 2 | 75 | 81 | 83 | 80 | 29.61 | 26 | s. s. w. | do |
| 3 | 71 | 100 | 89 | 84 | 29.50 | | w. s. w. | do |
| 4 | 72 | 89 | 77 | 79 | 29.46 | | n. | do |
| 5 | 60 | 86 | 70 | 72 | 29.54 | | n. w. | Clear. |
| 6 | 56 | 96 | 81 | 78 | 29 53 | | w. | Variable. |
| 7 | 66 | 86 | 72 | 75 | 29.48 | | e. | do |
| 8 | 57 | 85 | 73 | 72 | 29.61 | | n. e. | Clear. |
| 9 | 51 | 85 | 78 | 71 | 29.52 | | w. | do |
| 10 | 71 | 91 | 84 | 79 | 29.48 | | w. s. w. | Variable. |
| 11 | 71 | 97 | 86 | 85 | 29.48 | | s. w. | do |
| 12 | 72 | 100 | 84 | 85 | 29.49 | | w. n. w. | do |
| 13 | 75 | 99 | 90 | 88 | 29.50 | | w. s. w. | do |
| 14 | 70 | 100 | 87 | 86 | 29.56 | | w. | do |
| 15 | 74 | 78 | 77 | 76 | 29.53 | 30 | calm. | do |
| 16 | 55 | 86 | 75 | 72 | 29.56 | | w. s. w. | do |
| 17 | 62 | 86 | 74 | 74 | 29.51 | | s. e. | do |
| 18 | 59 | 88 | 80 | 76 | 29.57 | | e. n. e. | do |
| 19 | 59 | 92 | 82 | 78 | 29.54 | | n. e. | do |
| 20 | 60 | 95 | 85 | 80 | 29.61 | | s. s. w. | Clear. |
| 21 | 59 | 99 | 83 | 81 | 29.64 | | s. | do |
| 22 | 63 | 99 | 84 | 82 | 29.62 | | e. | do |
| 23 | 65 | 102 | 93 | 87 | 29.60 | | e. n. e. | do |
| 24 | 66 | 103 | 91 | 86 | 29.62 | | w. s. w. | do |
| 25 | 66 | 103 | 91 | 87 | 29.59 | | e. | do |
| 26 | 75 | 99 | 90 | 88 | 29.60 | | n. | Variable. |
| 27 | 70 | 78 | 71 | 73 | 29.61 | 1.00 | s. w. | do |
| 28 | 69 | 92 | 86 | 28 | 29.63 | | n. e. | do |
| 29 | 69 | 95 | 86 | 83 | 29.60 | | e. n. e. | do |
| 30 | 70 | 99 | 90 | 87 | 29.60 | | s. s. w. | do |
| 31 | 71 | 100 | 88 | 86 | 29.59 | | e. | Clear. |
| | | | | 80.6 | | 1.56 | | |

